# MNBDR: A Module Network Based Method for Drug Repositioning

**DOI:** 10.3390/genes12010025

**Published:** 2020-12-27

**Authors:** He-Gang Chen, Xiong-Hui Zhou

**Affiliations:** Hubei Key Laboratory of Agricultural Bioinformatics, College of Informatics, Huazhong Agricultural University, Wuhan 430070, China; 13247702278@163.com

**Keywords:** drug repositioning, module network, systems biology, random walk algorithm

## Abstract

Drug repurposing/repositioning, which aims to find novel indications for existing drugs, contributes to reducing the time and cost for drug development. For the recent decade, gene expression profiles of drug stimulating samples have been successfully used in drug repurposing. However, most of the existing methods neglect the gene modules and the interactions among the modules, although the cross-talks among pathways are common in drug response. It is essential to develop a method that utilizes the cross-talks information to predict the reliable candidate associations. In this study, we developed MNBDR (Module Network Based Drug Repositioning), a novel method that based on module network to screen drugs. It integrated protein–protein interactions and gene expression profile of human, to predict drug candidates for diseases. Specifically, the MNBDR mined dense modules through protein–protein interaction (PPI) network and constructed a module network to reveal cross-talks among modules. Then, together with the module network, based on existing gene expression data set of drug stimulation samples and disease samples, we used random walk algorithms to capture essential modules in disease development and proposed a new indicator to screen potential drugs for a given disease. Results showed MNBDR could provide better performance than popular methods. Moreover, functional analysis of the essential modules in the network indicated our method could reveal biological mechanism in drug response.

## 1. Introduction

The traditional process of drug development is particularly slow and costly, which usually takes 12–15 years and billions of dollars [1,2]. In addition, the rate of new drug candidates being Food and Drug Administration (FDA)-approved has been lessen although the levels of investments in pharmaceutical R&D remarkably increase [3]. At the same time, this philosophy of rational drug design that “one gene, one drug, one disease” paradigm overlooks the inherent complexity of diseases [4,5]. In this case, drug repositioning (or repurposing), which aims to identify novel disease indications for known safety and pharmacology approved-drugs, is very economical and efficient. Compared with traditional process of drug development, repositioning a drug may reduce the drug development period to 6.5 years and costs on average $300 million [6]. Therefore, drug repositioning should be “the primary strategy in drug discovery for every broadly focused, research-based pharmaceutical company” [1].

One of the seminal method is connectivity map (CMap) [7], and the assumption of it was that biological state could be described in terms of a genomic signature. They measured genome-wide transcriptional expression data across a multiple of cell lines treated with small drug molecules and matched these profiles with disease perturbation gene expression profiles to find new associations between drugs and diseases. Although it is difficult to interpret the meaning of predicted associations, the robust of disease signatures and the effectiveness of the method has been experimentally validated [8,9,10]. Inspired by the rationale behind the CMap method [7], numerous approaches for drug repositioning based on gene expression data and connectivity map have been developed. Zhang et al. [11] proposed a simple method to filter reference gene-expression profiles for the connection scoring scheme. In addition, more connection methods, such as eXtreme Sum score (XSum) [12], Xcos [13], was proposed to calculate the similarities between gene expression patterns of diseases and drugs. Iorio et al. [14] developed a drug repositioning method that constructed drug–drug similarity networks by comparing drug perturbation gene expression profiles. Saberian et al. [15] presented a novel machine learning-based method, which explored the anti-similarity between drugs and diseases to uncover new uses for drugs. However, these previous methods ignored the fact that both the pathogenesis of diseases and drug mode of action (MoA) have been revealed to be tightly connected with gene modules [16]. Chung et al. [17] developed Functional Module Connectivity Map (FMCM), using functional gene modules as disease signatures to build a connectivity map and its performance was superior to traditional signature-based drug-repurposing methods. Jia et al. [18] introduced a new framework incorporating the gene expression data and pathway analysis. They provided a new approach to explain the drug mode of action in a disease context. As we know, proteins, nucleic acids, and small molecules could form a dense network of molecular interactions in a cell, and there may be cross-talks among different functional modules in the cell [19]. Therefore, in drug repositioning, it may be helpful to consider the cross-talks among the function modules. However, as far as we know, there were no drug repositioning methods taking the cross-talks among modules into consideration.

To fill the gap, we present Module Network Based Drug Repositioning (MNBDR), a novel computational module for drug repositioning. We applied the module network to the field of drug repositioning for the first time and proposed two new indicators to evaluate the expression levels of modules and the score of drug-disease. First of all, dense clusters in PPI network were detected as modules. After that, as described in our previous study [20], the cross-talks among modules were identified by testing whether the connections among the genes in two modules were significantly high. Based on both the gene expression data of disease samples and the module network, Pagerank [21] algorithm was applied to rank the important modules in disease. Lastly, the gene expression data of the important modules in drug stimulation samples were further pooled together to calculate an overall connectivity score for each pair of drug–disease. In order to validate our method, we applied MNBDR in 19 cancer datasets and compared it with several popular signature-based drug repurposing methods. We showed that MNBDR performed better than previous methods. Finally, we analyzed the function of the important modules in our module network to investigate the biological meaning of our method.

## 2. Methods

### 2.1. Data Set and Preprocessing

The drug stimulation data was downloaded from The Library of Integrated Network-Based Cellular Signatures (LINCS) program (level 5; accession number: GSE70138), which contains 118,051 gene expression profiles from multiple human cultured cell lines (treatment and control) treated with 1827 bioactive small chemical molecules at varying concentrations. Each expression profile consists of moderated z-score value of 12,328 genes. LINCS team defined nine touchstone cell lines [22] and we used five cell lines (PC3, A375, HALE, MCF7 and HT29) which have the sample sizes more than 10,000. The pre-processing procedure for drug gene expression data was included in the Supplementary Materials and Figure S1.

Cheng et al. [12,13] showed that the majority of the compounds do not have sufficient therapeutic effects on cell lines. In our work, we applied the compound filtering procedure described by Cheng et al. [12] and used expression signal strength (ESS) to filter the drug stimulation samples. The details were described in Supplementary Materials and Figure S2. The microarray data for whole-genome mRNA expression of disease samples were downloaded from TCGA (The Cancer Genome Atlas) research network [23]. In order to generate more stable disease features, only data sets with at least three normal and three disease samples were considered for further processing. Lastly, we obtained a total of 3486 control samples and 60,460 disease samples from 19 cancer data sets. Then, for each cancer data set, we averaged disease and control samples and calculated the corresponding fold changes for all the genes.

The PPI data was derived from STRING database [24]. In order to reduce the false-positive interactions which are probably originated from prediction methods, we followed the strategy of Zhou et al. [25] and only the interactions with a confidence score of 770 or above were kept. In total, there were 36,619 unique interactions among 9474 proteins in the PPI.

### 2.2. Benchmark Standard

The golden standard of known drug indications were obtained from Quan et al. [26]. They identified the drug-indication relationships through Drug–Gene Interaction database (DGIdb) [27], Therapeutic Target Database (TTD) [28], and DrugBank [29]. Only the clinically supported or FDA-approved drug–disease relationships were used. In this study, we got a total of 2877 associations between 19 cancers and 477 drugs. All the drug and disease interactions used in this work were shown in Supplementary Table S1.

### 2.3. Construction of the Module Network

**Figure 1:**
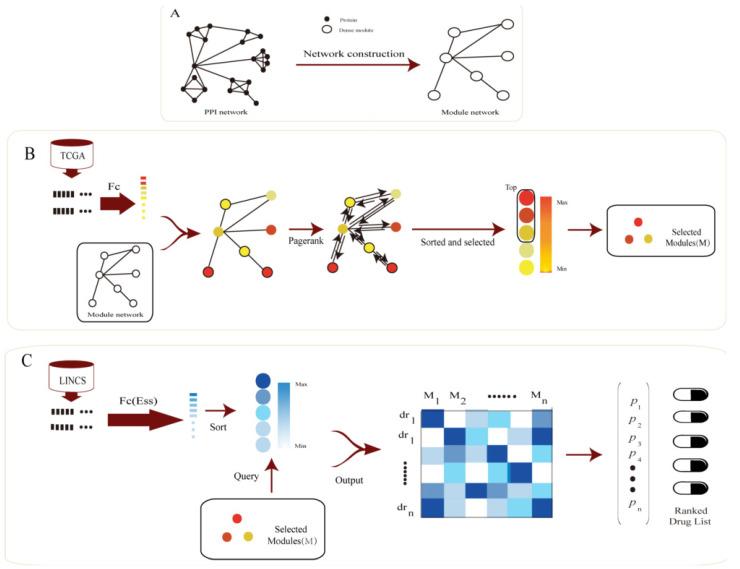
Pipeline of Module Network Based Drug Repositioning (MNBDR). (**A**) MNBDR detects dense modules in the PPI networks and captures the cross-talks among the modules by permutation test to form a module network. (**B**) Based on the module network and gene expression data of disease samples, MNBDR applies the Pagerank algorithm to rank the important modules in disease. (**C**) Using the important modules in disease and the gene expression data of drug stimulating samples, MNBDR applies a new indicator to infer potential associations between drugs and diseases.

To construct the module network, we adopted a similar strategy as our previous study [20]. First of all, we used MCODE [30] in Cytoscape [31] to detect dense clusters in the PPI network and only the clusters containing no less than 5 nodes were retained as modules. After that, for each pair of modules, the number of edges (PPI interactions) between the two modules was calculated. Then two random gene sets, which have the same number of genes with the two modules, were randomly selected, and the edges among the two random gene sets were counted. The random process was repeated 1000 times and the 1000 edge numbers were used as null distribution. Then, the *p*-value of the cross-talks among the two modules was calculated based on the null distribution. If the number of edges between two modules was significantly high (*p*-value < 0.01), then there was a cross-talk between the two modules. Finally, all the modules and cross-talks among these modules constituted the module network (Figure 1A).

### 2.4. Feature Space Transformation

For each disease (or drug), we mapped gene expression data from gene’s feature space to the module’s feature space. Taking breast cancer as an example, first of all, the fold-change of all the genes’ expression levels in the breast cancer samples and control samples was calculated. Then for all the *n* dense clusters (*M*_1_, *M*_2_, …, *M_i_*, …, *M_n_*) in PPI, the importance (*Imp_i_*) for module *M_i_* was calculated as follow:

$$Imp=\{\begin{array}{ccc} Fmax-Fmin & , & Fmax>0,Fmin<0\\ MAX(|Fmax|,|Fmin|) & , & others \end{array}$$

*Fmax* and *Fmin* are the maximum and minimum fold-change of all the genes in *M_i_*. At last, we obtained {*Imp*_1_, *Imp*_2_, …, *Imp_i_*, …, *Imp_n_*}, which can characterize the difference of the gene expression levels of all the modules in the disease.

### 2.5. Module Rank Based on Pagerank

We assumed that the modules with both important topological coefficient in the module network and significantly differential expression levels would be more essential in disease. We thus used network propagation algorithms to simulate cross-talks of functional modules, which was defined as follows:

$$P_{k}=\lambda WP_{k-1}+(1-\lambda )P_{0}$$

where *W* denotes a transition matrix that is the column normalization of the adjacency matrix. In our work, the nodes of the adjacency matrix are modules, and the edges are the connections among the modules in our module network. Here, *P*_0_ represents our initial, or prior, information of the modules. In this work, we set *P*_0_ as {*Imp*_1_, *Imp*_2_, *Imp*_3_, …, *Imp_n_*} of all the modules in the corresponding disease. As we know, if the propagation process repeated too much times, information will eventually spread out over the whole network and the local neighborhood of the important nodes will be missed [32]. Therefore, a damping factor *λ* (0 < *λ* < 1) was defined to avoid it. In this study, *λ* was set as 0.85, which was typical value for Pagerank [33].

### 2.6. Drug Prioritizing

Inspired by the normalized discounted cumulative gain (NDCG) [34], we proposed a new indicator *S* to evaluate the drug–disease score between each drug to a specific disease. The indicator is described as follows:

$$S=\sum_{i=1}^{n}\frac{V(i)}{|P(i)-i|+1}$$

For the top *n* modules (1st, 2nd, …, *i*th, …, *n*th) in disease progression, *V*(*i*) is the *imp* of the *i*th modules in drug response and *P*(*i*) is the position of the *i*th module in the ranked module list in drug response. That is, if the important modules in disease were also ranked on the top of the module list in drug response, a high score *S* would be obtained. At last, for each disease, all the drugs were prioritized based on *S*.

### 2.7. Evaluation Metrics

We used the area under the curve (AUC) of the receiver operator characteristic (ROC) measure to evaluate model performance. The ROC curve can be drawn with the true-positive rates (TPRs) and the false-positive rates (FPRs) at different cutoffs. TPR is the proportion of positive samples identified correctly among the total positive samples, while FPR is the ratio of misidentified negative samples accounting for all the negative samples. TPR and FPR are defined as follows:

$$TPR=\frac{TP}{TP+FN},\;FPR=\frac{FP}{TN+FP}$$

where *TP* and *TN* are the numbers of correctly identified positive and negative samples, and *FN* and *FP* are the numbers of positive and negative samples that are misidentified. At the same time, we also used AUC0.1, which is widely used in the field of drug repositioning [12], to evaluate our algorithm. Index AUC0.1 is the area under the curve measured of the ROC under the condition of *FPR* ≤ 0.1. It guarantees that indicator can focus on the early retrieval performance of the model by restricting *FPR*. It is essential because it is more realistic in drug repositioning when the candidate drug number is small. Thus, in this work, we applied AUC0.1 as the main index for module evaluation. To better compare model performance, we also used the average AUC (AvgAUC) of all the diseases as our evaluation index. To determine the statistical significance of the results, we calculated non-parametric *p*-value by performing 10,000 runs with random permutations of the drug–disease relation.

### 2.8. Assessment

To assess the performance of MNBDR, we compared the prediction results with several methods.

In order to investigate the impact of cross-talks between modules on prediction performance, we compared MNBDR with two other methods (Gene based method and Module based method). The Gene based method only used the gene’s fold-change to rank the genes and used the ranked gene list to screen drugs, which is similar with the traditional CMap. In the meanwhile, Module based method, which ranked the modules using the gene expression levels (without taking the module networks into calculation) and screened the drugs based on the ranked module list.

In addition, we also compared the performance of MNBDR with six classic connectivity methods (GASE0Score [35], GASE1Score [35], GASE2Score [35], KSScore [7], ZhangScore [11], XSumScore [12]).

At last, the performances of MNBDR and three latest published methods (LLE-DML [15], Cogena [18]) and EMUDRA [36]) were compared.

MNBDR and Module based method are described as above and have been implemented in Python package, which are freely available at https://github.com/nbnbhwyy/MNBDR. Details about other methods were available in Supplementary Material.

## 3. Results

### 3.1. Framework Overview

Based on the fact that cross-talks among functional modules could play important roles in drug response and disease progression, we proposed a computational method, which used module network to identify essential modules in disease progression and improve CMap drug screening strategy, to do drug repositioning. Figure 1 shows the pipeline of our method.

Our framework includes the following steps: (i) As PPI network exhibit a “scale-free” topology [37], communities exist in PPI network. The dense cluster in PPI may work together as a functional unit, we mined the communities in PPI as functional gene sets (denoted as modules in this work). After that, a permutation test was applied to identify the cross-talks among these modules (Method). As a result, we obtained 486 significant pairs among 116 modules, which were shown in Figure S3 and Supplementary Table S2. (ii) For each disease, the perturbation of the genes was calculated based on the gene expression data of disease samples and control samples, and then mapped the perturbation of the genes to the module’s space through an index *Imp* (Method) to obtain the initial score of the modules. Subsequently, we applied a network propagation algorithm to learn the topology information of the module network to refine the scores of the disease modules. In the propagation algorithm, λ is an important parameter and we adopted a typical value (0.85) [33]. In addition, we changed λ in the algorithm and found the result was robust (Figure S4). At last, we selected the *n* modules with the highest scores to characterize the corresponding disease. In this study, *n* is set to 15, which is about 10% of all the nodes in the module network. In order to validate the robustness of our model, we also varied n from 3 to 50 and found the result was stable, and our method could achieve the best performance with *n* = 15 (The details are included in Figure S5). (iii) For these modules, the perturbation scores after each drug’s stimulation was also calculated based on the gene expression data of the samples stimulated by the corresponding drug and the control sample (Method). Then, a new indicator *S* (Method) was proposed to evaluate the effect of each drug to the specific disease. Finally, all the drugs for each disease could be ranked based on the indicator.

### 3.2. Comparing with CMap

CMap is the most famous method to screen drugs using gene expression data [7]. Cheng et al. [12,13] have made a systematic assessment of CMap. In this work, we use this method as a benchmark (Gene based method). In addition, in order to validate the hypothesis that the cross-talks information among functional modules could facilitate drug repositioning, we compared our strategy (MNBDR), which integrating module networks and gene expression data to rank modules, with a simple expression ranking strategy which prioritizing modules based on expression data only (Module based method).

**Figure 2:**
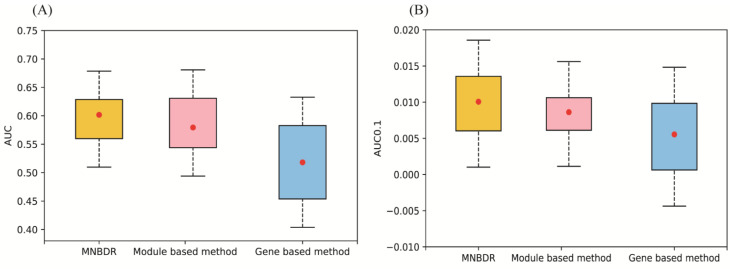
Performance of three different methods. (**A**) AUC. (**B**) AUC0.1.

Cancer is one of the most serious threats to human health and drug development for cancer is a big challenge [38]. Here, we applied our method in 19 cancer data sets to comparing the performance of MNBDR, Gene based method and Module based method. In this work, we adopted the same index (AUC, AUC0.1 and *p*-value) in a previous work to evaluate the performance of drug screen methods [12]. The detail result was shown in Table 1 and Figure 2.

The results showed that MNBDR had the best performance in the two indexes: AveAUC and AveAUC0.1 (FPR = 0.1, specificity higher than 0.9). In the meanwhile, the method based on modules performed better than the method using gene as features (the original method of CMap), which was consistent with the previous report [17]. In addition, this phenomenon also proved that the communities mined from PPI indeed were functional modules. Furthermore, because the main difference between our method and the method based on modules was that MNBDR using the cross-talks information in the module network, the better performance of our method validated the hypothesis of our strategy. At last, we also validated the performance by randomly permuted the drug–disease relations of the benchmark standard and calculated the *p*-values of the two indexes (Method). These *p*-values also proved the power of our method. The detail result was shown in Table S3.

### 3.3. Comparing with the Other Methods

The connectivity approach is essential for drug screen using gene expression data and a previous paper compared several connectivity approaches [39]. In order to evaluate the power of our method, we compared the performance of our method with all the five connectivity methods. From this result (Table 2), it can be seen that all the connectivity approaches can achieve a better performance than random methods (*p*-value < 0.01). Among them, XsumScore was the best. Apart from that, in the two indexes, MNBDR outperformed other connectivity methods.

In addition to comparing with traditional connectivity methods, we also compared our method with three latest published methods (LLE-DML [15] and Cogena [40] and EMUDRA [36]) that used gene expression data to screen drugs for diseases. The results shown in Table 2 indicated MNBDR was more effective than LLE-DML, Cogena, and EMUDRA. More importantly, the differences of AUC0.1 in the four methods are more obvious and AUC0.1 is very valuable for drug development [12], and we find that Cogena has similar performance with the Module based method. This can prove that our approach may be useful for different functional modules, which is valuable for further research. Moreover, LLE-DML achieves the second best performance. It performs as “black boxes” and it is very hard to investigate the important genes in the modules. In the meanwhile, our method could reveal the important modules in diseases, and could be used to investigate the biological mechanisms in disease progression and drug response.

### 3.4. Function Analysis of the Important Modules in Diseases

**Figure 3:**
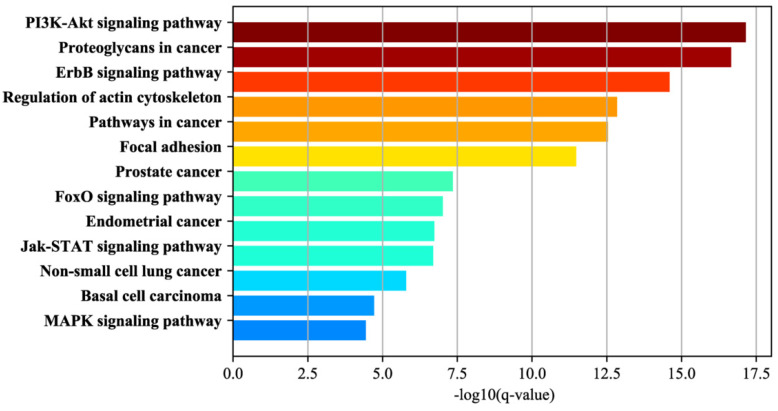
Functional annotation of the module genes.

We also investigated the function of the important modules to reveal the underlying mechanisms in disease and drug response. In our study, we selected the modules that are important in all the 19 cancers and set them as GCF (generalized cancer features). As a result, 15 modules were selected. Then, we used GSEA to analyze which pathways the genes, contained by GCF were involved in. Some enriched KEGG pathways for GCF genes were shown in Figure 3 and all the enriched pathways were shown in Supplementary Table S4.

Among the 47 significant pathways (FDR < 1.0 × 10^−4^), we found that “Pathways in cancer” was on the top, with an FDR of 2.89 × 10^−13^. What is more, many sub-pathways of “Pathways in cancer” were enriched, such as “MAPK signaling pathway”, “PI3K-Akt signaling pathway”, “FoxO signaling pathway”, “Proteoglycans in cancer”, “Jak-STAT signaling pathway”, “Regulation of actin cytoskeleton”, “Focal adhesion” and “ErbB signaling pathway”. In these sub-pathways, “MAPK signaling pathway” is reported to be essential for cancer-immune evasion in human cancer cells [41]. In addition, “PI3K-Akt signaling pathway” plays a major role not only in tumor development but also in the tumor’s potential response to cancer treatment [42]. Recent studies indicate that numerous components of the phosphatidylinositol-3-kinase (PI3K)/AKT pathway have more frequent amplification, mutation and translocation than any other pathway in cancer patients [43]. About “Proteoglycans in cancer”, the available evidence indicates both an up-regulation of ribosome production and changes in the ribosome structure might causally contribute to neoplastic transformation [44]. Forkhead box O (FOXO) transcription factors are involved in multiple signaling pathways and function as tumor suppressors in a variety of cancers [45]. Apart from this, there were also many pathways in specific cancers, such as “NON-Small cell lung cancer”, “Prostate cancer”, “endometrial cancer”, and “Basal cell carcinoma”.

In a word, module genes are significantly enriched with many cancer-related pathways.

### 3.5. Case Study in Breast Cancer

Breast cancer is one of the most common cancer and drug screen for breast is essential for the therapy [46]. As described above, MNBDR could achieve a good performance in breast cancer data set. The details of drugs identified by MNBDR for breast cancer are included in the Supplementary Table S5. Among the identified drugs, most of them are also supported by the literature, in addition to being confirmed by the benchmark data.

Romidepsin is predicted as an efficient drug for breast cancer by our method. In the meanwhile, Romidepsin is a histone deacetylase inhibitor treatment of adult patients with cutaneous T-cell lymphoma (CTCL) [47]. It modulates additional targets involved in cancer initiation and progression such as c-myc, Hsp90 and p53. Romidepsin has shown anticancer effects by induction of apoptosis, cell differentiation and cell cycle arrest, either alone or in combination [48,49].

Colchicine has been considered as one of the most effective medications for alleviating crystal-induced joint inflammation [50]. Inhibition of microtubule polymerization is the chief mechanism of action. Microtubules are among the main protein filaments that make up the cytoskeleton, which is crucial to the regulation of many activities [51]. To date, microtubule-targeting agents (MTAs) remain one the most reliable classes of antineoplastic drugs in the treatment of BC [52]. Based on this evidence, Colchicine which inhibits of microtubule polymerization may have a potential therapeutic effect on breast cancer and the experiment also got a certain degree of verification [53,54], and Colchicine is predicted as one of the most efficient drugs for breast cancer by MNBDR.

Ciclopirox is also one of the top rank drugs predicted by our method for treating breast cancer. This drug is a diterpene triepoxide that is able to suppression the cell growth of breast cancer [55,56].

All the results indicate our method could not only prioritize the drugs which have been approved, but also find the new adaptation disease for the old drugs.

## 4. Conclusions

As cross-talks among modules may be important in disease progression and drug response, we proposed a module network based drug repositioning (MNBDR) method. We used module network, which is based on a permutation test method, to describe the cross-talks among the modules in PPI, and then, using the gene expression data of disease samples and control samples, a network diffusion method was used to rank important modules in disease. After that, the important modules in each drug were also identified using gene expression data of samples stimulated by the drug. Finally, a new index, which could reveal whether the important modules in disease progression were also important in drug response, was proposed to evaluate the efficiency of a drug to the specific disease. We evaluated our method using gene expression data of more than 7000 samples from 19 different cancers obtained from TCGA, as well as measurements of around 118,051 drug instances LINCS databases. The results showed that MNBDR consistently outperformed the other methods in terms of not only AUC but also AUC0.1, which indicated the proposed method performed well when the effective drugs are on the top of the ranked lists. Function annotation of the genes in the modules shows our method indeed could capture the import genes in disease and drug response. In addition, case study in breast cancer showed our method could not only prioritize the drugs which have been approved, but also find the new adaptation disease for the old drugs.

In order to prevent overfitting, we did not train the model and we adopted typical values for the parameters in the model. As *n* (the number of important models) in the model is very important, we also used 10-fold cross-validation to select the best *n* in training set and compared the performance between our current strategy (*n* = 15) and the optimal model in 10-fold cross validation. We repeated the 10-fold cross-validation 10 times and the result was shown in Figure S6. We found that the result of optimal model is similar to our current strategy, which proves that our model does not have a data leakage problem. Of course, there are some drawbacks of our work. In this work, we only validated our method in cancer data sets. In fact, drug repositioning for other diseases are also valuable. In our future work, we will test our method in more diseases.

## Figures and Tables

**Table 1:** The performances of three methods on the 19 cancer datasets.

Method	AveAUC	*p*-Value	AveAUC0.1	*p*-Value
Gene based method	0.520	4.6 × 10^−6^	0.0055	2.7 × 10^−2^
Module based method	0.579	3.3 × 10^−69^	0.0086	2.6 × 10^−41^
MNBDR	0.602	6.2 × 10^−114^	0.0101	1.7 × 10^−80^

**Table 2:** Comparison results of all methods on the 19 cancer datasets.

Method	AveAUC	*p*-Value	AveAUC0.1	*p*-Value
GASE2Score	0.534	2.3 × 10^−14^	0.0065	8.7 × 10^−9^
GASE1Score	0.532	6.2 × 10^−13^	0.0063	4.9 × 10^−7^
GASE0Score	0.520	4.6 × 10^−6^	0.0055	2.7 × 10^−2^
ZhangScore	0.518	3.3 × 10^−5^	0.0055	2.7 × 10^−2^
XSumScore	0.548	8.2 × 10^−27^	0.0079	1.4 × 10^−27^
**MNBDR**	**0.602**	**6.2 × 10^−114^**	**0.0101**	**1.7 × 10^−80^**
LLE-DML	0.586	1.3 × 10^−81^	0.0086	2.6 × 10^−41^
Cogena	0.572	7.7 × 10^−58^	0.0080	2.3 × 10^−29^
EMUDRA	0.538	1.7 × 10^−17^	0.0058	1.1 × 10^−3^

The results of our method (MNBDR) are bolded.

## Data Availability

Publicly available datasets were analyzed in this study. This data can be found here: NCBI GEO: https://www.ncbi.nlm.nih.gov/geo/query/acc.cgi?acc=GSE70138, TCGA: https://portal.gdc.cancer.gov/. All source code in this study is available at https://github.com/nbnbhwyy/MNBDR.

## Supplementary Materials

The following are available online at https://www.mdpi.com/2073-4425/12/1/25/s1, Supplementary Figure S1—Drug data pre-processing pipeline. Supplementary Figure S2—Expression signal strengths of all compound expression profiles on five cell lines. Supplementary Figure S3—Module Interaction Network Based on PPI. Supplementary Figure S4—The performance of MNBDR with different parameters. Supplementary Figure S5—Performance of Drug–disease association prediction with top n modules as important modules (*λ* = 0.85). Supplementary Figure S6—The performance of our method using 10-fold cross validation. Supplementary Table S1—Drug-Indication relationships (benchmark standard). Supplementary Table S2—The genes involved in the Modules. Supplementary Table S3—The results of each cancer type. Supplementary Table S4—Functional annotation of module genes. Supplementary Table S5—The score of disease-drug pairs.

## References

[1] Tobinick E.L. (2009). The value of drug repositioning in the current pharmaceutical market. Drug News Perspect..

[2] Avorn J. (2015). The $2.6 Billion Pill—Methodologic and Policy Considerations. N. Engl. J. Med..

[3] Swinney D.C., Anthony J. (2011). How were new medicines discovered?. Nat. Rev. Drug Discov..

[4] Hopkins A.L. (2008). Network pharmacology: The next paradigm in drug discovery. Nat. Chem. Biol..

[5] Sams-dodd F. (2005). Target-based drug discovery: Is something wrong? define rational drug discovery programs. Drug Discov. Today.

[6] Nosengo N. (2016). Can you teach old drugs new tricks?. Nat. News.

[7] Molecules S., Lamb J., Crawford E.D., Peck D., Modell J.W., Blat I.C., Wrobel M.J., Lerner J., Brunet J., Subramanian A. (2006). The connectivity map: Using using gene-expression signatures to connect small molecules, genes, and disease. Science.

[8] Dudley J.T., Tibshirani R., Deshpande T., Butte A.J. (2009). Disease signatures are robust across tissues and experiments. Mol. Syst. Biol..

[9] Nevins J.R., Potti A. (2007). Mining gene expression profiles: Expression signatures as cancer phenotypes. Nat. Rev. Genet..

[10] Dudley J.T., Sirota M., Shenoy M., Pai R.K., Roedder S., Chiang A.P., Morgan A.A., Sarwal M.M., Pasricha P.J., Butte A.J. (2011). Computational repositioning of the anticonvulsant topiramate for inflammatory bowel disease. Sci. Transl. Med..

[11] Zhang S.-D., Gant T.W. (2008). A simple and robust method for connecting small-molecule drugs using gene-expression signatures. BMC Bioinform..

[12] Cheng J., Yang L., Kumar V., Agarwal P. (2014). Systematic evaluation of connectivity map for disease indications. Genome Med..

[13] Cheng J., Xie Q., Kumar V., Hurle M., Freudenberg J.M., Yang L., Agarwal P. (2013). Evaluation of analytical methods for connectivity map data. Biocomputing.

[14] Iorio F., Bosotti R., Scacheri E., Belcastro V., Mithbaokar P., Ferriero R., Murino L., Tagliaferri R., Brunetti-Pierri N., Isacchi A. (2010). Discovery of drug mode of action and drug repositioning from transcriptional responses. Proc. Natl. Acad. Sci. USA.

[15] Saberian N., Peyvandipour A., Donato M., Ansari S., Draghici S. (2019). A new computational drug repurposing method using established disease-drug pair knowledge. Bioinformatics.

[16] Xiong M., Li B., Zhu Q., Wang Y.X., Zhang H.Y. (2014). Identification of transcription factors for drug-associated gene modules and biomedical implications. Bioinformatics.

[17] Chung F.H., Chiang Y.R., Tseng A.L., Sung Y.C., Lu J., Huang M.C., Ma N., Lee H.C. (2014). Functional Module Connectivity Map (FMCM): A framework for searching repurposed drug compounds for systems treatment of cancer and an application to colorectal adenocarcinoma. PLoS ONE.

[18] Jia Z., Liu Y., Guan N., Bo X., Luo Z., Barnes M.R. (2016). Cogena, a novel tool for co-expressed gene-set enrichment analysis, applied to drug repositioning and drug mode of action discovery. BMC Genom..

[19] Spirin V., Mirny L.A. (2003). Protein complexes and functional modules in molecular networks. Proc. Natl. Acad. Sci. USA.

[20] Zhou X.H., Chu X.Y., Xue G., Xiong J.H., Zhang H.Y. (2019). Identifying cancer prognostic modules by module network analysis. BMC Bioinform..

[21] Page L., Brin S., Motwani R. (1999). The PageRank Citation Ranking: Bringing Order to the Web.

[22] Subramanian A., Narayan R., Corsello S.M., Peck D.D., Natoli T.E., Lu X., Gould J., Davis J.F., Tubelli A.A., Asiedu J.K. (2017). A Next Generation Connectivity Map: L1000 Platform and the First 1,000,000 Profiles. Cell.

[23] Koboldt D.C., Fulton R.S., McLellan M.D., Schmidt H., Kalicki-Veizer J., McMichael J.F., Fulton L.L., Dooling D.J., Ding L., Mardis E.R. (2012). Comprehensive molecular portraits of human breast tumours. Nature.

[24] Szklarczyk D., Gable A.L., Lyon D., Junge A., Wyder S., Huerta-Cepas J., Simonovic M., Doncheva N.T., Morris J.H., Bork P. (2018). STRING v11: Protein–protein association networks with increased coverage, supporting functional discovery in genome-wide experimental datasets. Nucleic Acids Res..

[25] Zhou H., Wong L. (2011). Comparative analysis and assessment of M. Tuberculosis H37Rv protein-protein interaction datasets. BMC Genom..

[26] Quan Y., Luo Z.H., Yang Q.Y., Li J., Zhu Q., Liu Y.M., Lv B.M., Cui Z.J., Qin X., Xu Y.H. (2019). Systems chemical genetics-based drug discovery: Prioritizing agents targeting multiple/reliable disease-associated genes as drug candidates. Front. Genet..

[27] Wagner A.H., Coffman A.C., Ainscough B.J., Spies N.C., Skidmore Z.L., Campbell K.M., Krysiak K., Pan D., McMichael J.F., Eldred J.M. (2016). DGIdb 2.0: Mining clinically relevant drug-gene interactions. Nucleic Acids Res..

[28] Qin C., Zhang C., Zhu F., Xu F., Chen S.Y., Zhang P., Li Y.H., Yang S.Y., Wei Y.Q., Tao L. (2014). Therapeutic target database update 2014: A resource for targeted therapeutics. Nucleic Acids Res..

[29] Law V., Knox C., Djoumbou Y., Jewison T., Guo A.C., Liu Y., MacIejewski A., Arndt D., Wilson M., Neveu V. (2014). DrugBank 4.0: Shedding new light on drug metabolism. Nucleic Acids Res..

[30] Pruitt K.D., Maglott D.R. (2001). RefSeq and LocusLink: NCBI gene-centered resources. Nucleic Acids Res..

[31] Paul Shannon A.M. (2003). Cytoscape: A Software Environment for Integrated Models of Biomolecular Interaction Networks. Genome Res..

[32] Cowen L., Ideker T., Raphael B.J., Sharan R. (2017). Network propagation: A universal amplifier of genetic associations. Nat. Rev. Genet..

[33] Halu A., Mondragón R.J., Panzarasa P., Bianconi G. (2013). Multiplex PageRank. PLoS ONE.

[34] Järvelin K., Kekäläinen J. (2002). Cumulated Gain-Based Evaluation in IR techniques. ACM Trans. Inf. Syst..

[35] Subramanian A., Tamayo P., Mootha V.K., Mukherjee S., Ebert B.L., Gillette M.A., Paulovich A., Pomeroy S.L., Golub T.R., Lander E.S. (2005). Gene set enrichment analysis: A knowledge-based approach for interpreting genome-wide expression profiles. Proc. Natl. Acad. Sci. USA.

[36] Zhou X., Wang M., Katsyv I., Irie H., Zhang B. (2018). EMUDRA: Ensemble of multiple drug repositioning approaches to improve prediction accuracy. Bioinformatics.

[37] Deeds E.J., Ashenberg O., Shakhnovich E.I. (2006). A simple physical model for scaling in protein-protein interaction networks. Proc. Natl. Acad. Sci. USA.

[38] Umar A., Dunn B.K., Greenwald P. (2012). Future directions in cancer prevention. Nat. Rev. Cancer.

[39] Lin K., Li L., Dai Y., Wang H., Teng S., Bao X., Lu Z.J., Wang D. (2019). A comprehensive evaluation of connectivity methods for L1000 data. Brief. Bioinform..

[40] Guney E., Menche J., Vidal M., Barábasi A.L. (2016). Network-based in silico drug efficacy screening. Nat. Commun..

[41] Sumimoto H., Imabayashi F., Iwata T., Kawakami Y. (2006). The BRAF-MAPK signaling pathway is essential for cancer-immune evasion in human melanoma cells. J. Exp. Med..

[42] Hennessy B.T., Smith D.L., Ram P.T., Lu Y., Mills G.B. (2005). Exploiting the PI3K/AKT Pathway for Cancer Drug Discovery. Nat. Rev. Drug Discov..

[43] Fresno Vara J.Á., Casado E., de Castro J., Cejas P., Belda-Iniesta C., González-Barón M. (2004). P13K/Akt signalling pathway and cancer. Cancer Treat. Rev..

[44] Montanaro L., Treré D., Derenzini M. (2008). Nucleolus, ribosomes, and cancer. Am. J. Pathol..

[45] Fu Z., Tindall D.J. (2008). FOXOs, cancer and regulation of apoptosis. Oncogene.

[46] Chung K. (2018). Rapid drug screen using 3D tumor organoids. Sci. Transl. Med..

[47] Yang L.P.H. (2011). Romidepsin: In the treatment of T-cell lymphoma. Drugs.

[48] Campas-Moya C. (2009). Romidepsin for the treatment of cutaneous T-cell lymphoma. Drugs Today.

[49] Robertson F.M., Chu K., Boley K.M., Ye Z., Liu H., Wright M.C., Moraes R., Zhang X., Green T.L., Barsky S.H. (2013). The class I HDAC inhibitor Romidepsin targets inflammatory breast cancer tumor emboli and synergizes with paclitaxel to inhibit metastasis. J. Exp. Ther. Oncol..

[50] Roberts W.N., Liang M.H., Stern S.H. (1987). Colchicine in Acute Gout: Reassessment of Risks and Benefits. JAMA J. Am. Med. Assoc..

[51] Niel E., Scherrmann J.M. (2006). Colchicine today. Jt. Bone Spine.

[52] Dumontet C., Jordan M.A. (2010). Microtubule-binding agents: A dynamic field of cancer therapeutics. Nat. Rev. Drug Discov..

[53] Sun Y., Lin X., Chang H. (2016). Proliferation inhibition and apoptosis of Breast Cancer MCF-7 cells under the influence of colchicine. J. BUON.

[54] Wang R.C., Chen X., Parissenti A.M., Joy A.A., Tuszynski J., Brindley D.N., Wang Z. (2017). Sensitivity of docetaxel-resistant MCF-7 breast cancer cells to microtubule-destabilizing agents including vinca alkaloids and colchicine-site binding agents. PLoS ONE.

[55] Li J., Liu R., Yang Y., Huang Y., Li X., Liu R., Shen X. (2014). Triptolide-induced in vitro and in vivo cytotoxicity in human breast cancer stem cells and primary breast cancer cells. Oncol. Rep..

[56] Shao H., Ma J., Guo T., Hu R. (2014). Triptolide induces apoptosis of breast cancer cells via a mechanism associated with the Wnt/β-catenin signaling pathway. Exp. Ther. Med..

