# Supplementary figures and images for: MNBDR: A Module Network Based Method for Drug Repositioning
Source: Genes (Basel). 2020 Dec 27;12(1):25. doi: 10.3390/genes12010025 (PMC7824496; doi:10.3390/genes12010025)

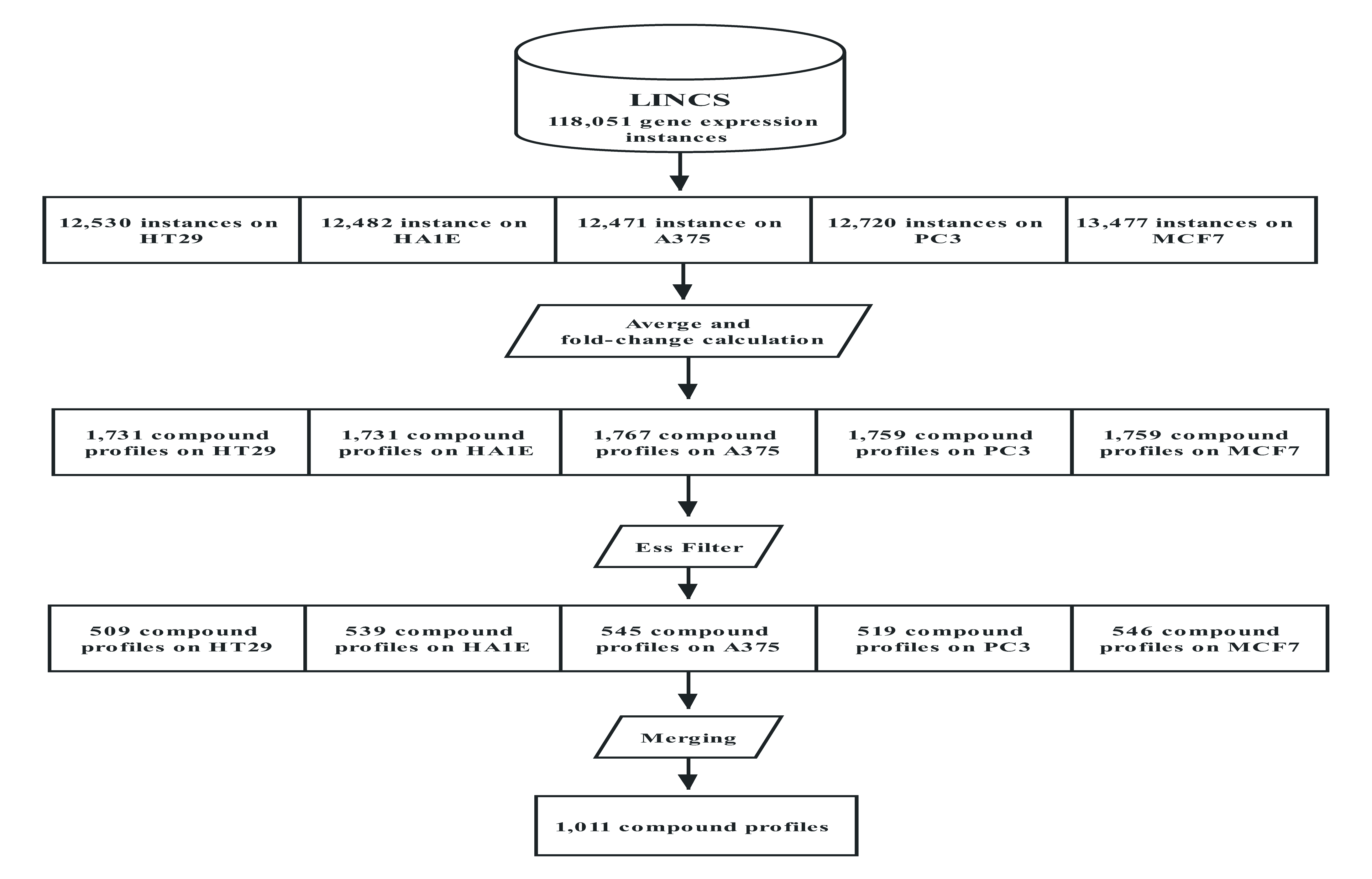

Supplement: Supplementary file 1 [file genes-12-00025-s001.zip › genes-12-00025-s001/genes-1013750-supplementary/Supplementary/FigureS1.tif]

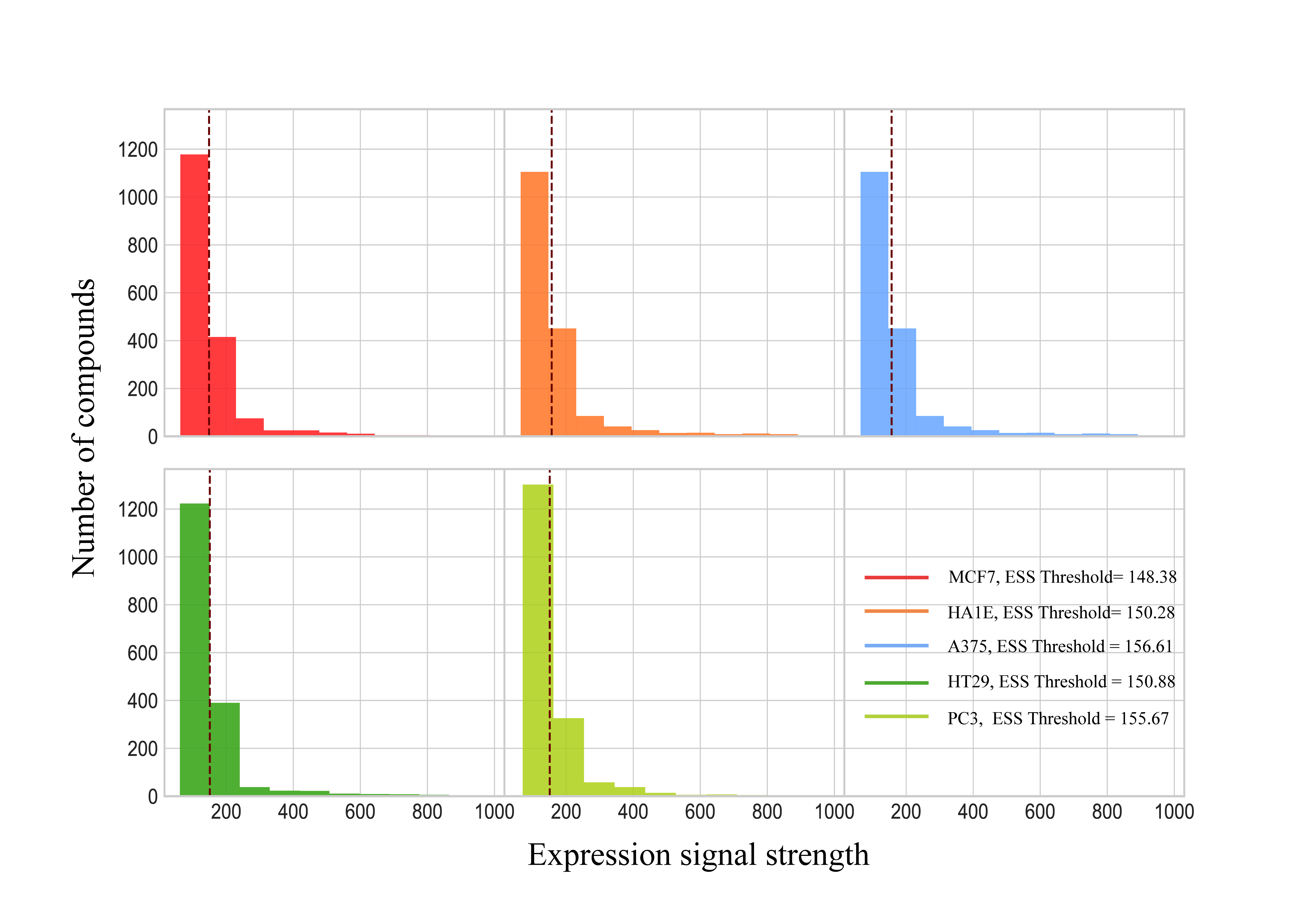

Supplement: Supplementary file 1 [file genes-12-00025-s001.zip › genes-12-00025-s001/genes-1013750-supplementary/Supplementary/FigureS2.tif]

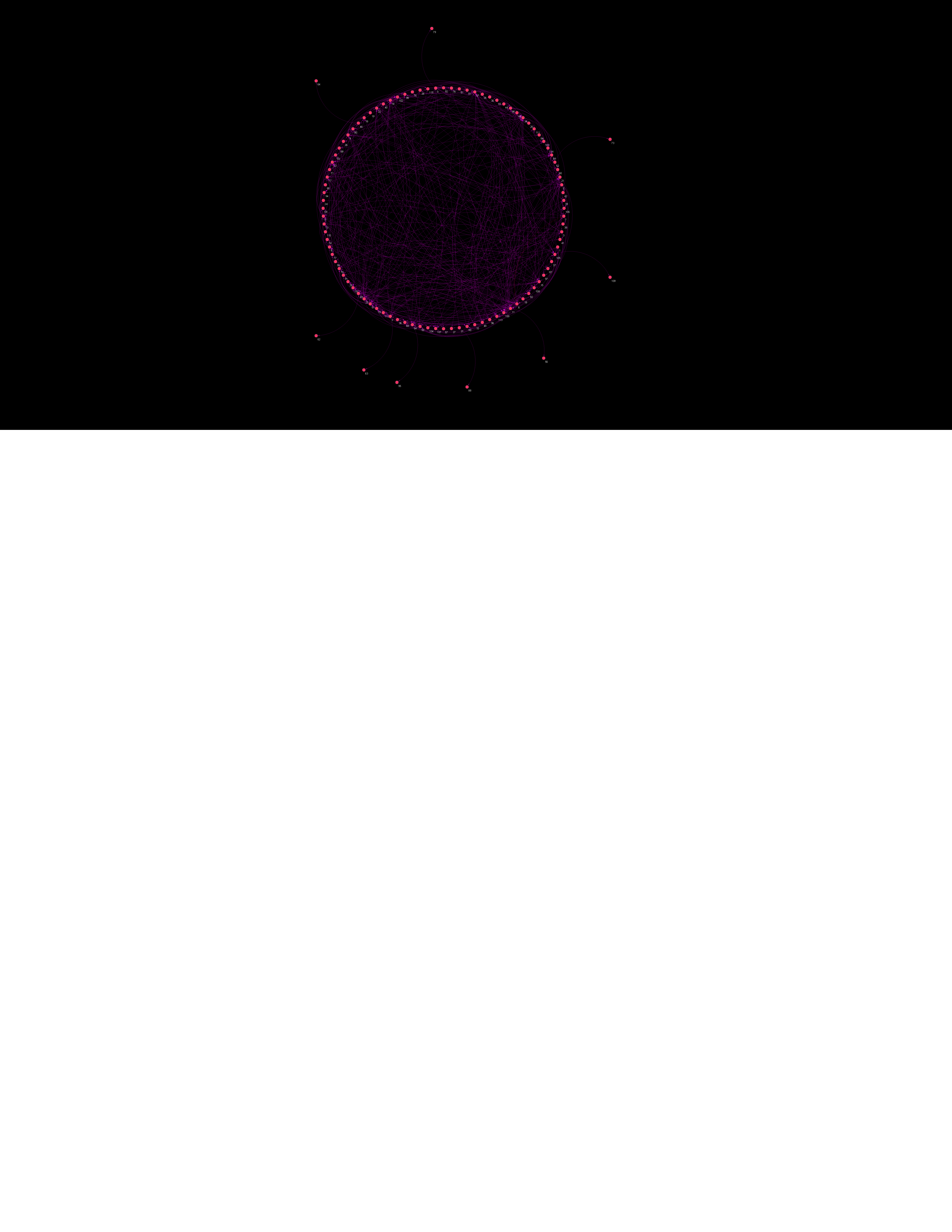

Supplement: Supplementary file 1 [file genes-12-00025-s001.zip › genes-12-00025-s001/genes-1013750-supplementary/Supplementary/FigureS3.tif]

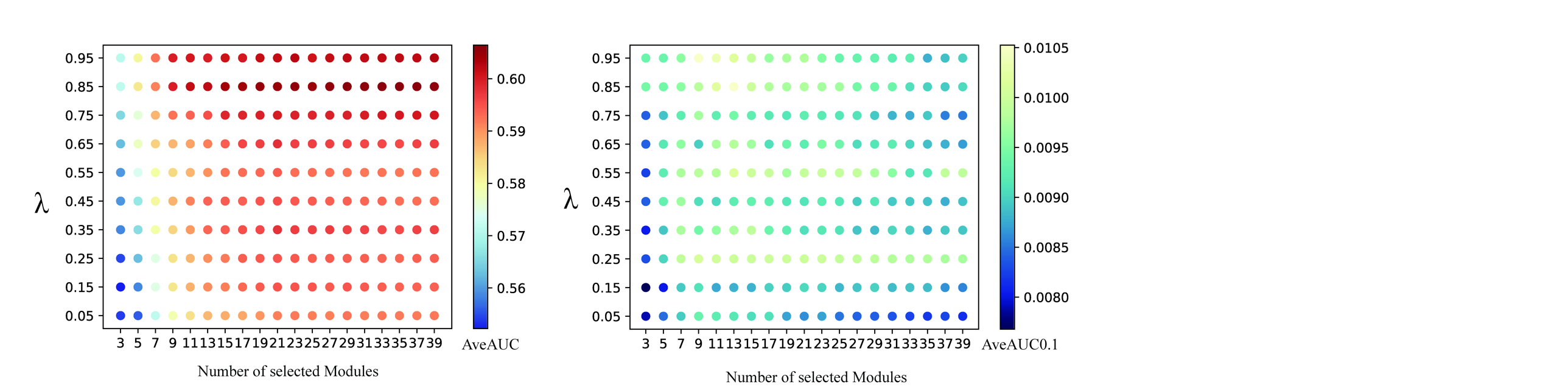

Supplement: Supplementary file 1 [file genes-12-00025-s001.zip › genes-12-00025-s001/genes-1013750-supplementary/Supplementary/FigureS4.tif]

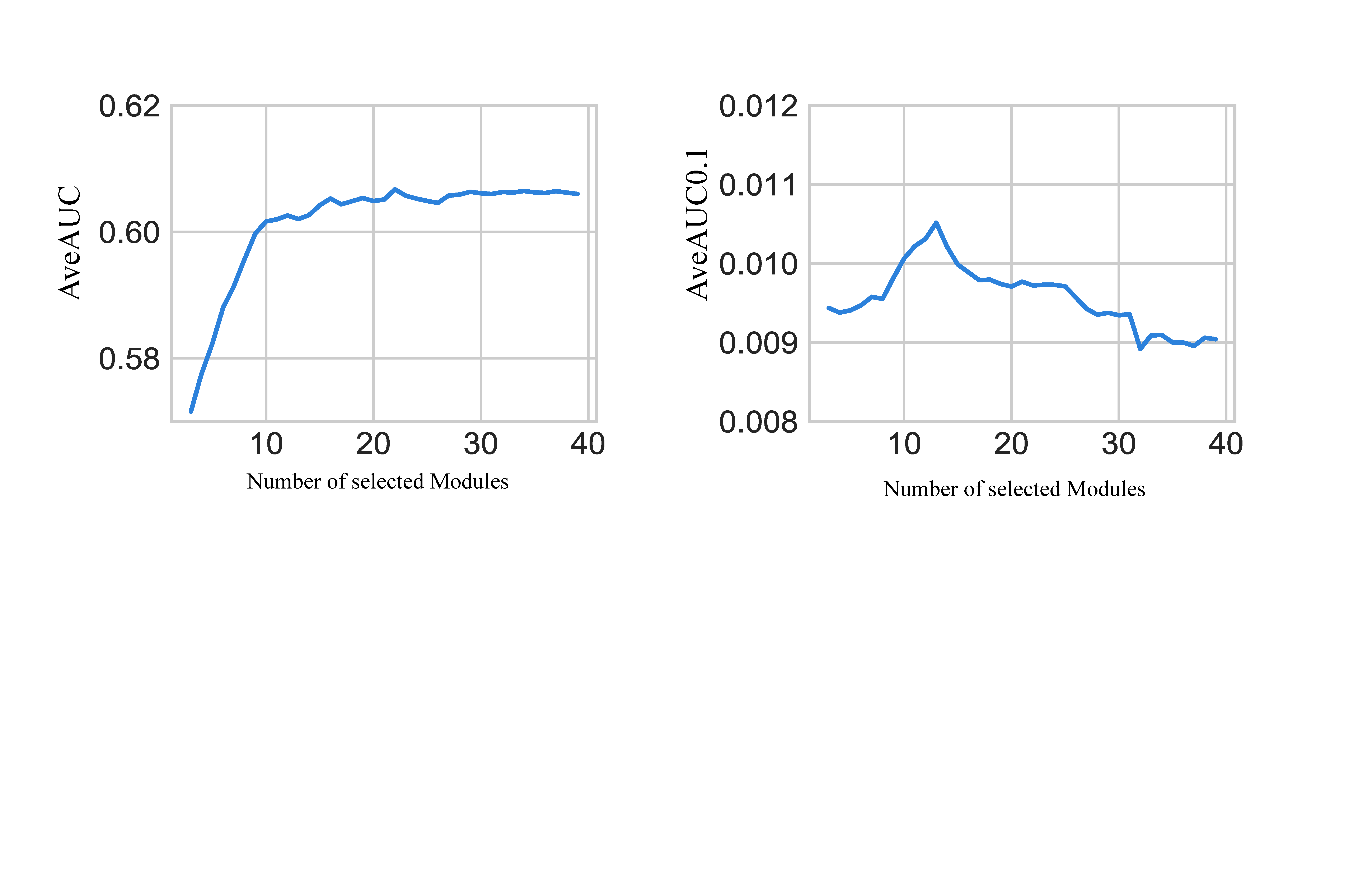

Supplement: Supplementary file 1 [file genes-12-00025-s001.zip › genes-12-00025-s001/genes-1013750-supplementary/Supplementary/FigureS5.tif]

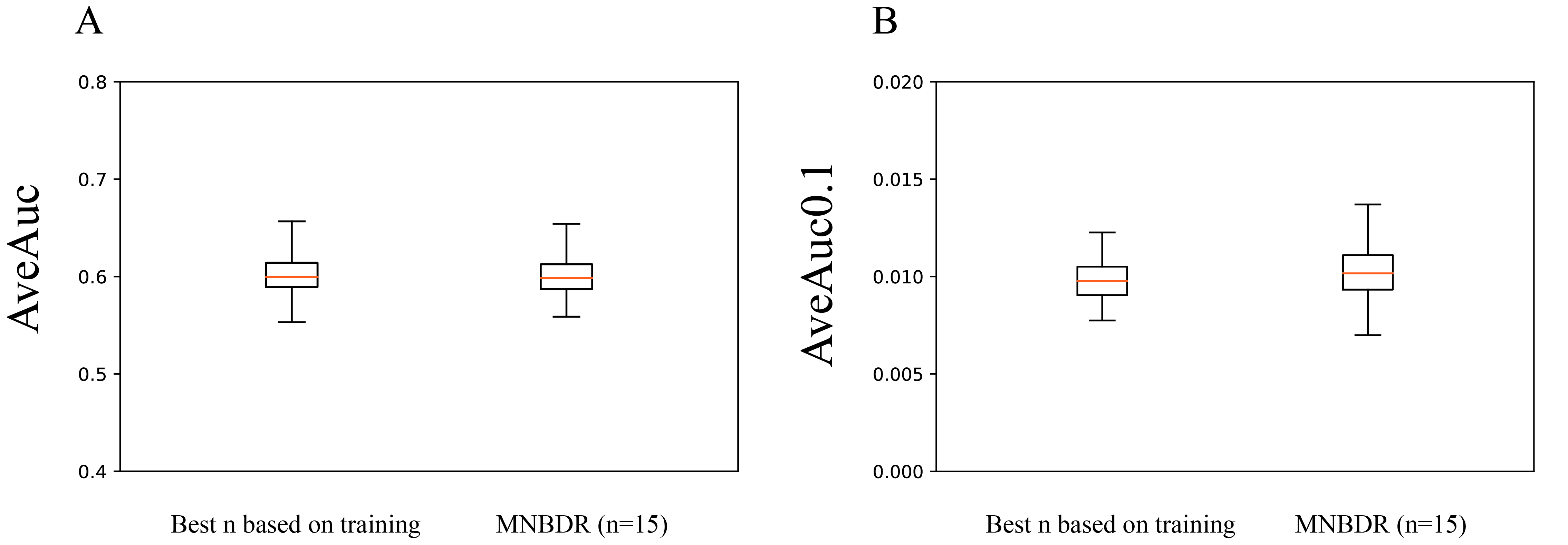

Supplement: Supplementary file 1 [file genes-12-00025-s001.zip › genes-12-00025-s001/genes-1013750-supplementary/Supplementary/FigureS6.tif]
