# Supplementary material for: MNBDR: A Module Network Based Method for Drug Repositioning
Source: Genes (Basel). 2020 Dec 27;12(1):25. doi: 10.3390/genes12010025 (PMC7824496; doi:10.3390/genes12010025)
Supplement: Supplementary file 1 [file genes-12-00025-s001.zip › genes-12-00025-s001/genes-1013750-supplementary/Supplementary/Supplementary material-Genes.doc]

MNBDR: A Module Network Based Method for Drug Repositioning

**He-Gang Chen^1^, Xiong-Hui Zhou^1,*^**

^1^ Hubei Key Laboratory of Agricultural Bioinformatics, College of Informatics, Huazhong Agricultural University, Wuhan 430070, China

^*^Correspondence: Correspondence should be addressed to X. Z. ([zhouxionghui@mail.hzau.edu.cn](mailto:zhouxionghui@mail.hzau.edu.cn))

Drug gene expression data

For all drug data, multiple instances of the same compound in the same cell line were averaged to generate cell line specific compound profiles. For each gene we calculated the corresponding fold change between the two types (treated vs. control). We then used expression signal strength (ESS) [1] to filter the cell line specific compound profiles (described in the later section) and after the filtering step, we kept 509, 539, 545, 519 and 546 compound profiles for HT29, HA1E, A375, PC3 and MCF7, respectively. The overall work flow is shown in Figure S[1](https://genomemedicine.biomedcentral.com/articles/10.1186/s13073-014-0095-1#Fig1).

**Figure. S1.** Drug data pre-processing pipeline

Other Methods used in this work

CMap [2] is based on a simple but intuitive idea that a drug would be effective if it counteract the effect of the disease on every single differentially expressed genes. The initial CMap approach utilized a non-parametric, rank-based Kolmogorov-Smirnov (KS) statistic to represent the anti-correlation of disease expression profiles and drug simulation profiles (We denoted it as Gene based method and used it as a control method to evaluate our algorithm). Based on CMap [2], Zhang et al. [3] presented a new framework, based on signed-rank statistic signed-rank statistic that provided effective control over false connections (ZhangScore). Cheng et al. [1] systematically evaluated the power of CMap and replaced KS statistics with a simple scoring algorithm called eXtreme Sum (XSum), which has a promising early retrieval performance. In this work, we denoted these three methods as Gene based method, ZhangScore, XSumScore respecitively.

Gene Set Enrichment Analysis (GSEA) team proposed a KS-like method which based on weighted Kolmogorov–Smirnov-like statistic (ES) [4]. The GSEA team provided three scoring schemes that implement the GSEAPreranked algorithm, we denoted these schemes as GASE0Score, GASE1Score, GASE2Score.

Jia et al. [5] developed Cogena (co-expressed gene-set enrichment analysis), a novel framework that based on module analysis. Cogena can be used to to explain coordinated changes within disease transcriptomes and identify drugs acting mechanistically within this framework (we denoted the method as Cogena).

Zhou et al*.* [6] designed an expression weighted cosine (EWCos) method to minimize the influence of the uninformative expression changes and developed an ensemble approach termed ensemble of multiple drug repositioning approaches (EMUDRA) to combine multiple matching scores approaches (we denoted the method as EMUDRA).

Saberian et al. [7] was directly influenced by the rationale behind the CMap and presented a novel machine learning-based method, which explored the anti-similarity between drugs and diseases to uncover new uses for drugs. Firstly, high-dimensional feature space of the gene expression data was mapped into a low-dimensional feature space by using Locally Linear Embedding (LLE) algorithm. Then, they used distance metric learning (DML) algorithm to find a suitable distance measure in the low-dimensional feature space such that the known disease–drug pairs got closer to each other. Finally, Euclidean distance between each disease–drug pair was calculated and the drugs were selected based on the distance (we denoted the method as LLE-DML).

All connectivity methods (GASE0Score, GASE1Score, GASE2Score, KSScore, ZhangScore, XSumScore) were implemented using R package RCSM [8]. Item LLE-DML was implemented using R package dml v1.1.0 ( Tan*g et a*l., 2014). The ProximityScore’ was implemented using Python package toolbox [10].

Method for Drug data filter

Expression signal strength (ESS) is a filtration Indicator for cell line-specific compound profiles [11]. For each drug, the ESS is defined as the sum of the absolute values of the fold changes of the top and bottom N features of the gene expression profile (N is set as 50 in this study). The cell line specific threshold for compound profile filtering is defined as following.

$$Ess \text{ }Threshold=median\text{ }Ess+1\times median\text{ }absolute\text{ }deviation$$

**Figure S2.** Expression signal strengths of all compound expression profiles on five cell lines.

**Figure S3.**   Module Interaction Network Based on PPI

**Figure S4.**   The performance of MNBDR with different parameters.

**Figure S5.**   Performance of Drug–disease association prediction with top n modules as important modules (λ=0.85)

**Figure S6.**   The performance of our method using 10-fold cross validation

**References**

1.	Cheng, J.; Yang, L.; Kumar, V.; Agarwal, P. Systematic evaluation of connectivity map for disease indications. *Genome Med.* **2014**, *6*, 1–8, doi:10.1186/s13073-014-0095-1.

2.	Molecules, S.; Lamb, J.; Crawford, E.D.; Peck, D.; Modell, J.W.; Blat, I.C.; Wrobel, M.J.; Lerner, J.; Brunet, J.; Subramanian, A.; et al. The Connectivity Map : Using. **2006**, *313*, 1929–1935.

3.	Zhang, S.-D.; Gant, T.W. A simple and robust method for connecting small-molecule drugs using gene-expression signatures. *BMC Bioinformatics* **2008**, *9*, 258, doi:10.1186/1471-2105-9-258.

4.	Subramanian, A.; Tamayo, P.; Mootha, V.K.; Mukherjee, S.; Ebert, B.L.; Gillette, M.A.; Paulovich, A.; Pomeroy, S.L.; Golub, T.R.; Lander, E.S.; et al. Gene set enrichment analysis: A knowledge-based approach for interpreting genome-wide expression profiles. *Proc. Natl. Acad. Sci. U. S. A.* **2005**, *102*, 15545–15550, doi:10.1073/pnas.0506580102.

5.	Jia, Z.; Liu, Y.; Guan, N.; Bo, X.; Luo, Z.; Barnes, M.R. Cogena, a novel tool for co-expressed gene-set enrichment analysis, applied to drug repositioning and drug mode of action discovery. *BMC Genomics* **2016**, *17*, 1–11, doi:10.1186/s12864-016-2737-8.

6.	Zhou, X.; Wang, M.; Katsyv, I.; Irie, H.; Zhang, B. EMUDRA: Ensemble of multiple drug repositioning approaches to improve prediction accuracy. *Bioinformatics* **2018**, *34*, 3151–3159, doi:10.1093/bioinformatics/bty325.

7.	Saberian, N.; Peyvandipour, A.; Donato, M.; Ansari, S.; Draghici, S. A new computational drug repurposing method using established disease-drug pair knowledge. *Bioinformatics* **2019**, 1–7, doi:10.1093/bioinformatics/btz156.

8.	Lin, K.; Li, L.; Dai, Y.; Wang, H.; Teng, S.; Bao, X.; Lu, Z.J.; Wang, D. A comprehensive evaluation of connectivity methods for L1000 data. **2019**, *00*, 1–12, doi:10.1093/bib/bbz129.

9.	Tang, Y. dml (2015) Distance Metric Learning in R. CRAN. R package version 1.1.0.

10.	Guney, E.; Menche, J.; Vidal, M.; Barábasi, A.L. Network-based in silico drug efficacy screening. *Nat. Commun.* **2016**, *7*, 1–13, doi:10.1038/ncomms10331.

11.	Cheng, J.; Xie, Q.; Kumar, V.; Hurle, M.; Freudenberg, J.M.; Yang, L.; Agarwal, P. Evaluation of analytical methods for connectivity map data. *Pacific Symp. Biocomput.* **2013**, 5–16.
